# An Online Cross-Sectional Research Domain Criteria (RDoC) Positive Valence Assessment Study With Patients With a Severe Mental Illness: Methodological Evaluation

**DOI:** 10.2196/86998

**Published:** 2026-11-11

**Authors:** Marit Broer, Marjolein E A Barendse, Laura van den Brink-Steinmann, Anne L Snijders, Marisha N Meijer, Sofie Paludanus, Monique van der Weijden-Germann, Robert M Bilder, Roel Ophoff, Neeltje E M van Haren

**Affiliations:** 1 Department of Child and Adolescent Psychiatry/Psychology, Erasmus University Medical Center, Wytemaweg 8, Rotterdam, 3015CN, The Netherlands; 2 Department of Developmental and Educational Psychology, Institute of Psychology, Leiden University, Leiden, The Netherlands; 3 Brain Research and Innovation Centre, Ministry of Defence, Utrecht, The Netherlands; 4 Department of Psychiatry, Brain Centre, University Medical Centre Utrecht, Utrecht, The Netherlands; 5 Force Health Protection, Netherlands Armed Forces, Ministry of Defence, Doorn, The Netherlands; 6 Semel Institute for Neuroscience and Human Behavior, University of California, 760 Westwood Plaza, Los Angeles, CA, United States, 1 3108259989; 7 Department of Psychology, College of Letters & Science, University of California, Los Angeles, CA, United States; 8 Department of Human Genetics, David Geffen School of Medicine, University of California, Los Angeles, CA, United States

**Keywords:** positive valence systems, reward processing, bipolar disorder, schizophrenia, online study

## Abstract

**Background:**

Abnormal reward processing is considered a core feature of severe mental illness (SMI). Deficits in this domain can have severe implications for daily functioning. The Online Positive Valence Study aimed to assess reward processing in bipolar disorder and schizophrenia using the National Institute of Mental Health (NIMH) Research Domain Criteria (RDoC) Positive Valence Systems (PVS) framework in an online task-based study. Recruitment and study adherence fell short of expectations.

**Objective:**

This paper critically evaluates the methodology used and shares the lessons learned from conducting an online task-based study of reward processing in a sample of patients with SMI.

**Methods:**

Using a cross-sectional design, the Online Positive Valence Study measured reward processing using 3 reward tasks, the monetary incentive delay task (MID), effort expenditure of rewards task (EEfRT), and a social and coin version of the Bandit task. Participants were eligible if they were 18 years or older. They were recruited mainly via email from 2 completed prospective cohorts. From 2023, the initial recruitment goal was no longer followed. Recruitment strategies and task performance are evaluated.

**Results:**

The sample consisted of 319 participants: 186 patients (58.3%), 81 first-degree relatives (25.4%), and 52 controls (16.3%). Multinomial logistic regression analyses revealed participants who were additionally called were more than 4 times more likely to respond “no” (odds ratio [OR] 4.57, 95% CI 3.12 to 6.70, *P*<.001) and more than twice as likely to respond “yes” (OR 2.75, 95% CI 1.93 to 3.92, *P*<.001) versus no response compared with those who received only emails. Personal salutations in invitation mails reduced the likelihood of participation compared to nonpersonal salutations (OR 0.31, 95% CI 0.20 to 0.47, *P*<.001). Analyses of covariance (ANCOVAs) and linear mixed effects models (LLMs) revealed task performance was comparable between in-lab and online groups. Online participants showed longer reaction times on the MID task (*β*=20.43, SE=2.55, *t*_309.10_=8.02, *P*<.001) and lower win-stay rates on both Bandits (Bandit Coin: *β*=–0.55, 95% CI –0.68 to –0.42, SE=0.06, *t*_306.0_=–8.73, *P*<.001, η² *P*=.14; Bandit Social: *β*=–0.65, 95% CI –0.80 to –0.49, SE=0.08, *t*_307.2_=–8.54, *P*<.001, η² *P*=.14).

**Conclusions:**

For the first time, we performed a remote online task-based assessment of the PVS domain in patients with SMI, and we learned important lessons along the way. Future studies should carefully consider recruiting from existing cohorts; hire full-time dedicated personnel; and use personal approaches to recruitment, like phone calls, adjusting recruitment to the target population(s), and making participation technically as simple as possible. Attrition may be reduced by shortening the duration of participation and/or adding gamification. This study offers guidance for future research in online cognitive assessment in patients with SMI.

## Introduction

Technology is an indispensable part of contemporary health care and research. Since the COVID-19 pandemic, the use of technology in psychological care has grown tremendously [1,2]. Remote health care was a necessity during lockdowns and social distancing, even more so given the rapid increase of (mental) health problems during the pandemic. Consequently, research into the efficacy of remote health care has increased in recent years, showing that remote or online assessment and treatment can be equally reliable and effective as in-person treatment [3-6]. This growing evidence base not only supports the use of remote methods in clinical practice but also provides new opportunities for scientific research, since online studies can reach many more eligible participants than traditional in-lab studies. Task-based cognitive experiments allow for a more fundamental understanding of the brain and can be used for early identification of functional and cognitive decline. However, little is known about the validity of online task-based assessment completed by patients with a severe mental illness (SMI) [7]. In addition to the general advantages of saving time and avoiding commuting costs, individuals with SMI might find remote participation particularly helpful since they might suffer from active symptoms such as social avoidance or paranoia. Several recent studies have used online task-based assessments with patients with SMI to measure cognitive functions such as memory, attention, and inhibition and have found them to be feasible [8-13]. To our knowledge, we are the first to perform a remote online task-based assessment of the Positive Valence Systems (PVS) domain with patients with SMI.

The National Institute of Mental Health (NIMH) introduced the Research Domain Criteria (RDoC) framework to address a growing concern about the validity of the *Diagnostic and Statistical Manual of Mental Disorders* (*DSM*) and to allow for a more transdiagnostic and neurobiological approach to psychopathology [14,15]. The framework identifies the PVS as one of 6 major domains of human neurobehavioral functioning. The PVS is responsible for responses to positive motivational stimuli or contexts, including subconstructs of reward responsiveness, reward learning, and reward valuation [16] and can also be described as reward processing. Research suggests that impairments in reward processing might be a core feature in several psychiatric disorders, including bipolar disorder [17,18] and schizophrenia [19,20]. People with bipolar disorder show state-dependent patterns—with hyperactivation in the orbitofrontal cortex and ventral striatum during mania but reduced or normalized activation during euthymic or depressive phases [21-23,24]—yet scientific evidence is quite inconsistent. Conversely, schizophrenia is more uniformly associated with hypoactivation in the ventral striatum, anterior cingulate cortex, and insula, especially in patients with negative symptoms, such as apathy or anhedonia [21,25,26]. A transdiagnostic approach could result in a clearer view of reward processing deficits in psychiatric disorders, since they often have similar symptoms like anhedonia or psychosis [27,28]. Deficits in this domain could have several implications in daily life (eg, decreased motivation to engage in social activities or make healthy lifestyle choices). This study attempted to remotely measure PVS functioning in people with bipolar disorder or schizophrenia, unaffected siblings, and control participants using an online task-based study design. The goal of the Online Positive Valence Study was to develop, validate, and complete online screening of PVS and, in combination with the in-lab magnetic resonance imaging (MRI) study, to derive response profiles from the PVS tasks that possess the greatest concurrent validity with respect to individual differences in neural circuit function. Unfortunately, recruitment and study adherence fell short of expectations. This goal of the Online Positive Valence Study could therefore not be reached.

Here, the methods and procedures of the Online Positive Valence Study are described in detail, after which we examined our experiences and effort to raise recruitment numbers, with the main goal to support future task-based online studies. In addition, we explored whether performance on the reward tasks was comparable online versus in lab, as meaningful recommendations are only relevant if the remote assessments produce results consistent with laboratory procedures. The goal was to make future task-based online studies more successful by sharing our experience and to explore task performance comparability between online and in-lab participant groups.

## Methods

### Participant Characteristics

A total of 319 persons started participation in the Online Positive Valence Study, including 194 patients with bipolar disorder, 3 patients with schizophrenia, 81 first-degree relatives, and 54 control participants. The average age was 56.64 (SD 12.43) years, and 64.6% (206/319) of the sample was female. Regarding educational attainment, 0.3% (1/319) of participants had less than a secondary education, 8.2% (26/319) had completed secondary education, 7.2% (23/319) had a vocational tertiary education, 53% (169/319) had a professional tertiary education, and 25.4% (81/319) had a university education. Educational level was missing for 6% (19/319) of participants.

### Sampling Procedures

Participants were recruited from 2 existing cohorts: Dutch Bipolar Study (DBS) [29,30] and Genetic Risk and Outcome of Psychosis (GROUP) [31] using convenience sampling. The 2 cohorts combined comprised more than 5000 adult participants, of which approximately 1000 had schizophrenia; 1750 had bipolar disorder; 1000 were first-degree relatives; and 1500 were healthy, unaffected, control participants. The cohort studies were performed at the University Medical Center Utrecht (UMCU) between 2004 and 2015. All participants from DBS or GROUP who gave permission to be contacted for future research were eligible for recruitment for the current MRI or Online Positive Valence Study (from hereon, “MRI Study” and “Online Study”). Participants who declined participation in the MRI study were asked to consider the Online Study. The aim was to enroll at least 3500 participants from DBS and GROUP. Data collection took place from June 2021 to July 2024. The research team for the Online Study consisted of 1 research assistant for about 16 hours per week, who was occasionally supported by a student assistant, a part-time postdoctoral researcher for supervision, and the principal investigators. During data collection, the research assistant was responsible for recruitment, technological support, and monitoring of the Online Study. The recruitment strategy is visualized in Figure 1, where every block represents a manual action from the research team. Following the General Data Protection Regulation (GDPR), until participants gave permission for their personal information to be transferred to the Erasmus Medical Center, all correspondence with them originated from the UMCU. Eligible participants were sent a recruitment email in which they were informed about a new study. In the early phase of recruitment, participants were also called; however, this quickly turned out to be unsustainable due to the time it took and the research team who were committed to sending emails only. Participants were first asked permission to transfer their personal information from the UMCU to the Erasmus Medical Center. Once they approved, they received an invitation email via our data capture system, GemsTracker, with more details about the study, a link to the information letter, informed consent form, and a hyperlink to the Online Study. When setting up the Online Study, it was not yet possible to insert an individual’s name in the invitation email because of privacy regulations. People who wanted to participate in the study therefore received this email with a nonpersonal salutation, namely “Dear participant.” The research team believed more people would actually click on the link in the invitation email and start participation when they were personally saluted, which is also supported by the literature [32]. From May 2022 onward, it was possible to insert a personal name in GemsTracker without it registering in the system, thus without breaking GDPR guidelines. From January 2023 onward, recruitment for the Online Study was put on hold, since it was clear the recruitment target would not be reached and the MRI Study was in need of more personnel for recruitment and data collection. The research team called as many eligible participants as possible to offer them the in-lab MRI study, and recruitment for the Online Study was limited to participants that declined participation in the MRI study.

**Figure 1.**
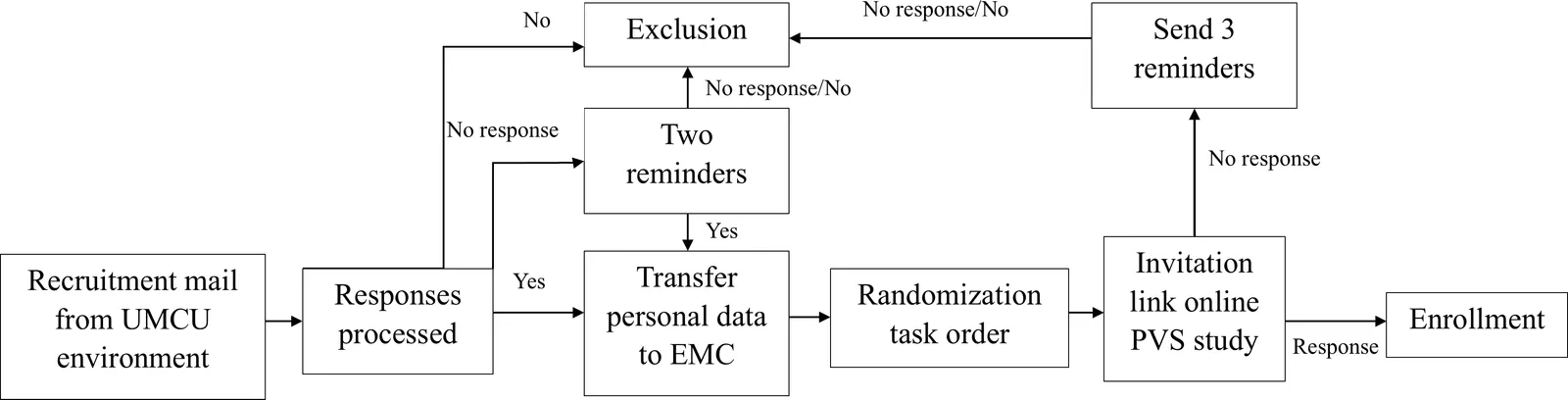
Flowchart of enrollment or exclusion of participants during the recruitment phase of Online Positive Valence Study. EMC: Erasmus Medical Center; PVS: Positive Valence Systems; UMCU: University Medical Center Utrecht.

### Measures and Covariates

To inventory clinical symptoms, the Altman Self-Rating Mania (ASRM) [33] was used to measure mania symptoms, the 8-item Patient-Reported Outcomes Measurement Information System (PROMIS) depression scale [34] was used to measure depressive symptoms, and the psychosis subscale of the Behavior and Symptom Identification Scale 24 (BASIS-24) [35] was used to measure psychotic symptoms. It also included questionnaires on the participants’ current demographic and clinical situation, response to reward, medication use, and motivational behavior. Parallel to the Online Study, there was an in-lab MRI Study with largely overlapping instruments (see Table S2 in Multimedia Appendix 1). To collect information on reward behavior, the Positive Valence Systems Scale (PVSS) [36] and the Temporary Experience of Pleasure Scale (TEPS) [37] were used. A self-constructed questionnaire was used to collect information on medication use and demographic variables. The Online Study (and MRI Study) comprised 3 tasks, each measuring different components of the PVS, namely the monetary incentive delay (MID) task [38] capturing reward responsiveness, the Effort Expenditure for Rewards Task (EEfRT) [39] that assesses reward valuation, and the Bandit task to assess reward learning. Specifications for the tasks were made by the investigators with special input from Katia Harle at the University of California San Diego for the Bandit tasks. An overview of the interviews and questionnaires used in the MRI study can be found in Table S2 in Multimedia Appendix 1.

### MID Task

The MID task was intended to measure anticipation of reward. For this task, each trial begins with a cue displayed for 500 milliseconds. This cue indicates whether the participant has the chance to gain or lose points (a circle represents a “win” trial, a square represents a “loss” trial) or if no points are involved (a triangle indicates a “neutral” trial). For win and lose trials, a single horizontal line within the circle or square indicates 75 points, while 2 horizontal lines indicate 300 points. After the cue, a fixation cross appears for a variable duration (6 s minus the durations of the cue and target stimulus). Next, participants must respond as quickly as possible to a target stimulus, a black star, which is displayed on the screen. They press a button box using their dominant hand. Feedback is then provided for 1500 milliseconds, showing whether their response was correct or incorrect (in green or red text, respectively) and the result of the trial: points earned (+75/+300), points lost (–75/–300), or no change (+0). The feedback also displays their cumulative score at that point in the task. Participants need to respond to the target stimulus within a specific time limit, which is personalized for each individual. This “target duration” is determined based on the average of the 16th and 17th slowest reaction times during 24 baseline trials conducted before the main task. The time limit is adjusted after each trial: If the participant’s correct response rate is <66%, 20 milliseconds are added; if it is ≥66%, 10 milliseconds are subtracted, provided the time limit remains between 150 milliseconds and 400 milliseconds. The task includes 60 trials, with 12 trials for each condition.

Participants of the MRI Study also completed a scanner version of the MID task, with the order of the in-scanner and out-of-scanner versions randomized across participants. To ensure engagement, participants with a nonresponse rate of 30% or higher will be excluded from the analysis. Outcome parameters extracted from this task entail the mean reaction time over all trials and difference in average reaction time (RT) between reward (combining small and large rewards) and neural trials.

### Effort Expenditure for Rewards Task

The EEfRT evaluates how participants assess effort versus reward. In this task, participants decide between an “easy” task that offers a smaller reward (100 points) or a “hard” task with a larger reward (ranging from 125 to 400 points) during each trial. The easy task requires 30 button presses within 7 seconds using the dominant hand’s index finger, while the hard task requires 100 button presses within 21 seconds using the nondominant hand’s pinky finger. Before each trial, the probability of receiving the reward (either 50% or 88%) is shown, along with the reward value for the hard task. Each trial begins with a 1-second fixation period, followed by a 5-second window for participants to choose between the easy or hard task. During this decision period, details about the reward probability and hard task reward magnitude are displayed. If the participant does not decide within 5 seconds, the computer randomly assigns the easy or hard task. After this choice period, the word “Ready” is shown for 1 second, signaling the start of the task. Participants then perform their chosen task, and upon completion, feedback is provided on whether they successfully finished within the time limit and whether they received a reward. The goal of the EEfRT is to accumulate as many points as possible within a 15-minute timeframe.

Trials where the participant did not choose between the 2 tasks in time (ie, forced trials) will be discarded. EEfRT data from a participant will only be analyzed if there are at least 20 nonforced trials. Outcome parameters retrieved from this task entail the overall percentage of hard-task choices and the difference in percentages of hard-task choices between the 2 probabilities.

### The 3-Armed Bandit Task

The 3-armed Bandit task was used to measure reward learning. Participants played two versions of this task: (1) the Coin Bandit task and (2) the Social Bandit task.

The 3-armed Coin Bandit task consists of 20 rounds, each with 16 trials, and is designed to investigate core processes of reward learning and decision-making. During each round, participants see 16 tokens displayed horizontally at the top of the screen. Their task is to assign each token to 1 of 3 arms by pressing the keys 1, 2, or 3 on the keyboard. After assigning a token, feedback is given: A green token indicates earning 1 coin, while a red token means no points were earned. The reward rates for each arm are independent and randomly determined at the start of each round using a beta distribution (α=*β*=2). The accumulated green and red tokens under each arm remain visible throughout the round. Participants have 20 seconds to assign each token. If they fail to respond within this time frame, the system prompts with “Are you still there?” Responding to this question restarts the current round, while no response ends the entire task. Participants are instructed beforehand to identify the arm with the highest reward rate in each round and strive to maximize their total points across all rounds.

The 3-armed Social Bandit task mirrors the structure of the 3-armed Coin Bandit task but replaces coins with compliments as rewards. It is designed to investigate social reward learning. Each round involves 3 virtual participants who are fictional but presented as real. These virtual players are depicted with a photograph taken from a public database along with an initial and age. Before starting the task, all participants complete a 10-item personality questionnaire. They are told that their responses were evaluated by up to 60 virtual players who could choose to give a compliment (eg, “You are honest”) based on the participant’s answers or opt to pass if they preferred not to give a compliment. In reality, the personality questionnaire does not affect the compliments, and participants are informed of this deception during the debriefing. The goal of the task is to maximize the number of compliments received across all rounds.

Bandit data from a participant are only analyzed if there are at least 16 completed rounds (decided separately for the coin and social version). Incomplete rounds will be discarded. Outcome measures are calculated separately for each version of the Bandit task and consist of the total score, the win-stay rate, lose-shift rate, and the learning rate. The win-stay rate is defined as the percentage of times a participant chooses the same arm after being rewarded by that arm in the previous trial, averaged across all rounds. The lose-shift rate is defined as the percentage of times a participant chooses a different arm after not being rewarded by an arm in the previous trial, averaged across all rounds. The learning rate is the linear slope of the likelihood of choosing the best arm as a factor of the trial number. This is calculated for the first one-half of all rounds as most learning takes place then and the slope is likely to get nonlinear in the second half of the runs. The best arm is the one that has the highest reward rate in that round.

### Data Collection

The research assistant built the local infrastructure to support the Online Study. Inquisit software (2020) [40] was used for the reward tasks, which is a software platform developed by Millisecond Software for designing and administering psychological tests and experiments. Participation was limited to a laptop or PC, not a mobile device, since a keyboard was necessary for the tasks. The infrastructure of the Online Study is shown in Figure 2. Clicking on the link in the invitation email brought participants to the GemsTracker website [41], the preferred data capture tool at the Erasmus Medical Center at that time. Limesurvey [42] stored the actual content of the Online Study in the form of “objects” (ie, the informed consent form, questionnaires, link to the tasks in Inquisit, and the debriefing). These “objects” are modular, code-based structures that can hold text or questions and allow for different kinds of inputs, such as radio buttons, an open text field, or just a “continue”button. The LimeSurvey objects containing the content were presented through GemsTracker. The invitation email included a link to a GemsTracker web page directing participants to a LimeSurvey object, where they provided informed consent after reading the information letter. Following informed consent, participants were asked to complete questionnaires (1 h) regarding medication use, demographic information, psychotic and mood symptoms, and response to reward and motivational behavior. The study questionnaires were divided across 4 LimeSurvey objects, with presentation order remaining constant across participants. The next 3 LimeSurvey objects each included a link to Millisecond, the web page at which the participant could download and run the Inquisit software to do the cognitive reward tasks (20 min per task). The order of the tasks was randomized. Participants received instructions for the download and were informed that they could pause their participation and resume at a later time. However, once a task was started, it was not possible to pause, so they were asked to finish the task before taking a break. The first task always included a brief instruction to make sure the software worked and participants understood the controls. The Inquisit tasks were programmed in such a way that, after finishing a task, the participant was automatically redirected back to the LimeSurvey object to continue participation (see Figure 2). Once a participant was on the page where they could play a task, they were instructed to click on a link that opened a new window to the Inquisit software to do the task. On the page with this link, there was a question whether they had played the game. This was done since participants would return to this page after finishing the task. After they responded to the question with a “yes,” they could click “next,” and they were guided to the next page (ie, Limesurvey object). They were explicitly asked to only respond “yes” to this question when they were done playing the task. As an incentive, participants were told they could earn up to €5 (US $5.60 as of October 14, 2026) per task based on their performance. After completing the tasks, participants were briefed about the fact that the reward they earned was in reality not dependent on their performance. Total assessment time was, on average, 2 hours. Participants were told a research assistant would check their participation, and after completion of all questionnaires (€5) and cognitive tasks (3 × €5), they would receive €20 (US $22.39 as of October 14, 2026) in gift cards within a week.

**Figure 2.**
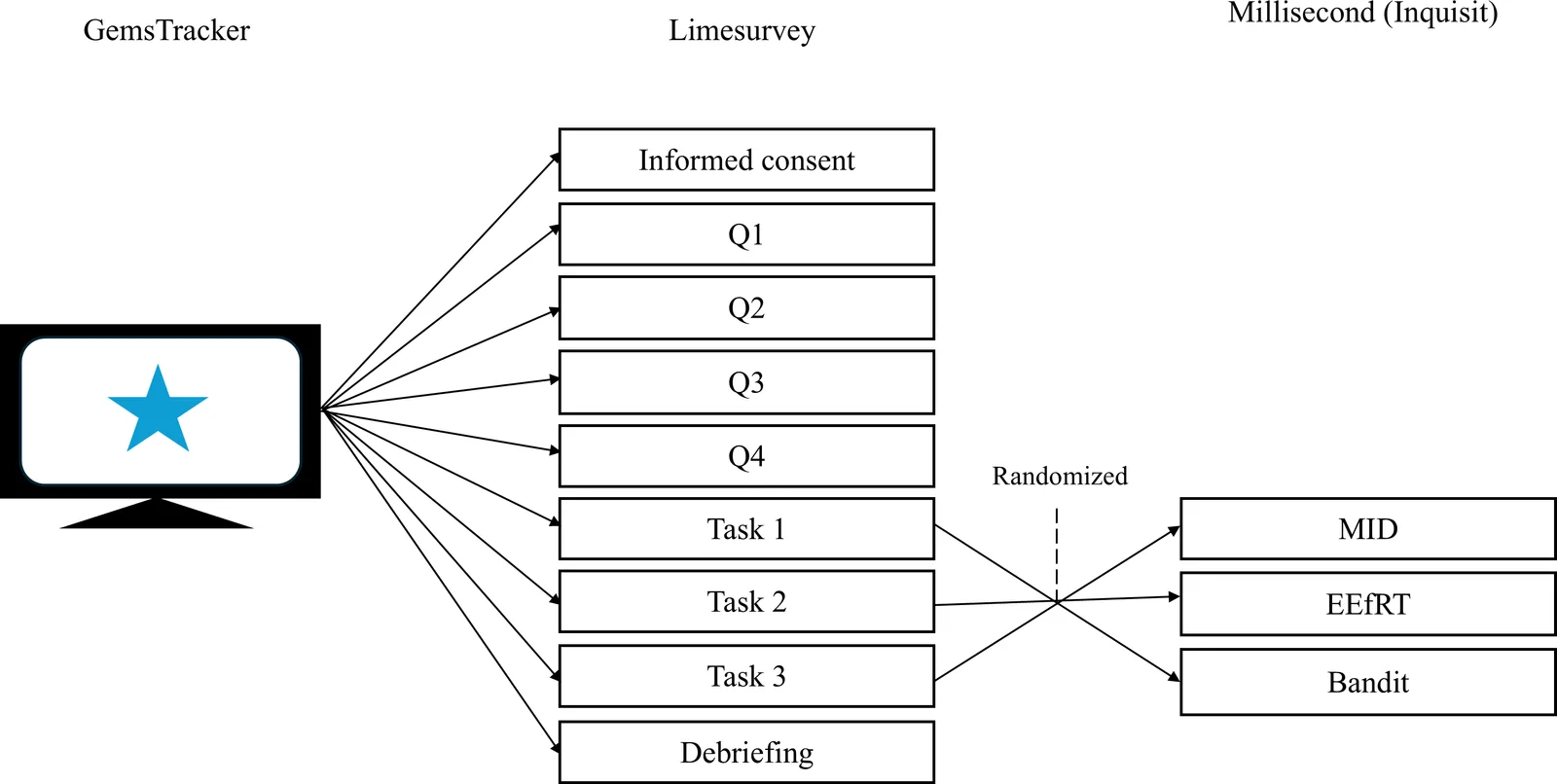
Schematic overview of the infrastructure of the online positive valence study, where GemsTracker is the logistical data capture tool, Limesurvey holds all objects hosted by GemsTracker and holds hyperlinks to Millisecond (Inquisit) that direct participants to the reward tasks; participants are directed back to Limesurvey objects when finished with a task and back to GemsTracker when finished with a Limesurvey object and are directed to the next Limesurvey object by GemsTracker. EEfRT=effort expenditure for rewards task; MID: monetary incentive delay; Q: questionnaire set.

### Data Diagnostics

Assumptions were tested prior to hypothesis testing. Data were not independent, as there were family members in the sample. Normal distribution of the outcome variables was assessed by visual inspection of violin plots and interpretation of skewness and kurtosis. The winstay rates of both Bandits (Coin and Social) were skewed (–1.8 and –2.0, respectively). The variables were winzorized to 0.1 SD away from the largest nonoutlier, which resulted in a decrease of skewness (–1.4 and –1.6, respectively) that the authors found acceptable. Multicollinearity was assessed curing a correlation matrix in R Studio using base R. The highest correlation was between the lose-shift rates of the Bandit Coin and Social (*r*=0.69) and was acceptable. Homoscedasticity was tested using the Levene test [43]. This assumption was violated for the difference in reaction time between a neutral and reward cue (MID), mean reaction time (MID), the lose-shift rates (Bandit Coin and Social), and the slope of best arm (Bandit Coin). Due to the number of violations, robust statistical tests were used for all variables of interest to handle violations of assumptions better.

We performed nonparametric missing completely at random (MCAR) tests in R studio using the MissMech package (version 1.0.4) [44] to test for the missing at complete random assumption.

Finally, to check for systematic bias for the participants who dropped out, we compared the online participants who completed all tasks with the online participants who did not complete all tasks using *t* tests for continuous variables (ie, age and psychopathological symptoms), a *χ*^2^ test for sex, and a Fisher exact test for educational level.

### Analytic Strategy

#### Effect of Recruitment Strategies

The effect of personalized recruitment strategies was assessed using multinomial and logistic regression analyses in R Studio (version 4.5.1) [45]. More specifically, the effect of a personal salutation in the invitation mail versus a nonpersonal salutation was assessed by analyzing the number of signed informed consent forms using a logistic regression analysis while also accounting for age, sex, and group. Personal salutation groups were based on invitation date: Participants receiving the mail before May 2022 received no personal salutation, whereas those receiving it after May 2022 did. In addition, the effect of additional phone contact was assessed by analyzing how many participants responded to participation (either yes or no) and how many participants did not respond between participants who were contacted through emails only and participants with whom the research team had spoken to on the phone at least once in addition to the standard emails. Telephone data were abstracted based on a contact log, where all contact with participants was registered during the course of recruitment and participation. This comparison was done by conducting multinomial regression analysis while accounting for age, sex, and group.

#### Task Performance in Lab Versus Online

To analyze differences in the dependent variables between online and in-lab participants, linear mixed effects models (LMMs) and analyses of covariance (ANCOVAs) were used. An overview of the statistical tests and standard error corrections per dependent variable, due to violated assumptions (see the Data Diagnostics section), can be found in Table 1.

Equivalence testing was conducted to explore group comparability on continuous variables using two 1-sided *t* test procedures implemented in the R package TOSTER (version 0.8.6) [46,47]. Equivalence was evaluated using the Cohen *d* with prespecified bounds of ±0.30. For categorical variables, equivalence between groups was tested using *χ*^2^ tests of independence. For the dichotomous variable sex, phi (φ) was reported as the effect size index. For educational level, the Cramér V was used. Equivalence bounds were set at *φ*=0.10 and V=0.10. Equivalence was suggested when the *χ*^2^ test was nonsignificant (*P*>.05) and the observed effect size fell below the prespecified bound, with the 90% CI for the effect size contained within that bound. Educational level contained cell frequencies <5, for which the Fisher exact test was used instead of the *χ*^2^ test. For psychotic and depressive symptoms, equivalence testing cannot be done between the in-lab and online study, as different instruments were used to assess these symptoms. The groups compared in this manuscript were not randomized, making equivalency highly unlikely. Therefore, these analyses were not intended to establish formal equivalence but rather to provide an indication of the extent to which task performance was comparable across settings.

**Table 1.** Overview of statistical tests and standard error corrections per dependent variable.

Statistical tests	Test	Robust SE correction
MID^a^		
Mean corrected reaction time overall	LMM^b^	Satterthwaite approximation
Difference in reaction time between neutral and reward trials	ANCOVA^c^	Huber-White estimate
EEfRT^d^		
Total percentage hard task choices	ANCOVA	N/A^e^
Difference in percentage hard task choices for different probabilities	LMM	N/A
Bandit		
Coin		
Total points	LMM	N/A
Slope best arm	ANCOVA	Huber-White estimate
Win-stay rate	LMM	N/A
Lose-shift rate	LMM	Satterthwaite approximation
Social		
Total points	LMM	N/A
Slope best arm	ANCOVA	N/A
Win-stay rate	LMM	N/A
Lose-shift rate	ANCOVA	Huber-White estimate

a MID: monetary incentive delay.

b LLM: linear mixed effects model.

c ANCOVA: analysis of covariance.

d EEfRT: Effort Expenditure for Rewards Task.

e N/A: not applicable.

Random intercepts were used to adjust for family relationships between participants when necessary. After (incomplete) data points were removed per task (for details on this procedure, see Multimedia Appendix 1), outliers of more than 3 SD were winsorized to 0.1 SD away from the largest nonoutlier. For differences in reaction time between a reward and neutral trial (MID), total percentage hard choices (EEfRT), slope best arm (Bandit Coin), slope best arm (Bandit Social), and lose-shift rate (Bandit Social), the random intercept to capture family relationships in the models did not explain any variance; thus, for these variables, performance was analyzed using ANCOVA. Assumptions were checked. A log-root transformation was done on the win-stay rate of the Bandit Coin and Bandit Social to correct for nonnormality. Robust standard error corrections were done when the assumption for homoscedasticity was violated, specifically for mean corrected reaction time overall (MID), difference in reaction time between a reward and neutral trial (MID), slope best arm (Bandit Coin), lose-shift rate (Bandit Coin), and lose-shift rate (Bandit Social). The Huber-White estimate [48,49] was used for ANCOVA, and the Satterthwaite approximation [50] was used for LMM. All analyses were done in R Studio (version 4.5.1) using the lmerTest (version 3.1‐3) [51], sandwich (version 2.0‐1) [52], and clubSandwich (version 0.6.1) [53] packages. It is important to note that participants were not randomized to the Online Study or in-lab MRI Study nor to any of the recruitment strategies. As such, the results are exploratory and should be interpreted with great caution. The comparative analyses are included in this paper to support the narrative of online versus in-lab task-based experiments.

### Ethical Considerations

The Online Positive Valence Study was approved by the Medical Ethics Committee Erasmus Medical Center on November 9, 2020 (MEC-2020‐0855). The committee concluded that there was no infringement of the physical or psychological integrity of the participants; thus, the study was not subject to the Medical Research Involving Human Subject Act (Wet Mengebonden Onderzoek [WMO]). Informed consent was given online before study procedures started. Eligible participants were asked to read this page carefully and either give consent and proceed or stop and exit the online environment used for the study. Collected data were pseudoanonymized, only research staff had access to personal data of the participants, and these data were not shared with other parties. After completion of the study, participants received €20 in gift cards.

The in-lab MRI Study was approved by the Medical Ethics Committee Erasmus Medical Center on February 18, 2020 (MEC-2019‐0384). This study adhered to the regulations of the WMO. Eligible participants were called about the study. After expressing interest, they received a participant information letter and were given sufficient time to consider participation. On the date of the visit, they were informed of their rights and that participation was voluntary. Once a trained research assistant found the participant able to make an informed decision, the participant signed their informed consent form and received a copy. During the tasks, participants were told that the amount of money they would receive in gift cards depended on their performance. After completing the study, they were informed that this was not the case and that the amount was fixed regardless of performance. All participants received €40 (US $44.78 as of October 14, 2026) in gift cards after completing the study.

No images nor tables included in the manuscript nor supplementary materials contain information that could enable the identification of individual participants or users.

## Results

### Participant Flow

Figure 3 is the Journal Article Reporting Standards (JARS) participant flowchart, and Figure 4 is a more detailed flowchart of all recruitment phases of the Online Study. Figure 5 shows the recruitment timeline of the Online Study. From the 1252 initial emails that were sent, 556 (556/1252, 44.4%) participants consented to their personal data being migrated to the Erasmus Medical Center and were sent a link to participate in the Online Study. A total of 332 participants enrolled in the Online Study, of whom 165 participants completed all study procedures. Figure 5 shows an increasing number of eligible participants who received an email about the Online Study. There were 2 periods (October 2021 and October 2022) in which the research assistant had sent a large number of emails. The number of participants that consented to data migration and enrolled increased slowly but more steadily over time. From 2023 onward, no new participants were approached for the Online Study, resulting in a flattening of the curve of eligible participants. Figure 6 shows that the first study procedures were answered more often than they were left unanswered. When the tasks began, this reversed, and an increasing number of participants left the procedures unanswered. This indicates a sharp rise in dropouts when participants were asked to play the reward tasks. Participants who completed all tasks were compared with the participants who did not complete all tasks (see Table S7 in Multimedia Appendix 1 for a full overview). The participants who did not complete all tasks were significantly older (*t*_291.01_=4.35, 95% CI 3.27 to 8.67, *P*<.001) and had different educational backgrounds (*P*=.04) compared with the participants who completed all tasks. Patients who did not complete all tasks had higher scores on mania symptoms than patients who completed all tasks (*t*_176.18_=2.18, *P*=.03, 95% CI 0.07 to 1.36). Nonpatients (ie, relatives and controls) who did not complete all tasks had higher scores on psychotic symptoms than nonpatients who completed all tasks (*t*_78.18_=2.38, *P*=.02, 95% CI 0.08‐0.95). In the Limesurvey task objects, participants were asked to first click the hyperlink to Millisecond and execute the task, after which participants would answer the question “did you play the task?” with a “yes” and click “next” to go to the next Limesurvey object and continue participation. However, a considerable portion (133/332, 40.1%) of participants responded to the question with “yes” and clicked “next” without having executed the task for at least 1 of the 3 tasks.

Information on demographics and clinical symptoms from the online participants and in-lab participants can be found in Table S1 in Multimedia Appendix 1. The MCAR test was nonsignificant for both the online participants (*P*=.55) and the in-lab participants (*P*=.58). This implied that there were no statistically significant differences in the missing value patterns across the variables in each group. The online and in-lab participant groups statistically differed in distribution of sex (χ^2^_1_=11.0, *P*<.001) and in research group distribution (χ^2^_2_=11.8, *P*=.003, V=0.13, 90% CI 0.06 to 1.00) and were not equivalent in age; the online participants were significantly older than those in the in-lab study. More detailed information on differences between groups can be found in Table S1 and Table S7 in Multimedia Appendix 1.

**Figure 3.**
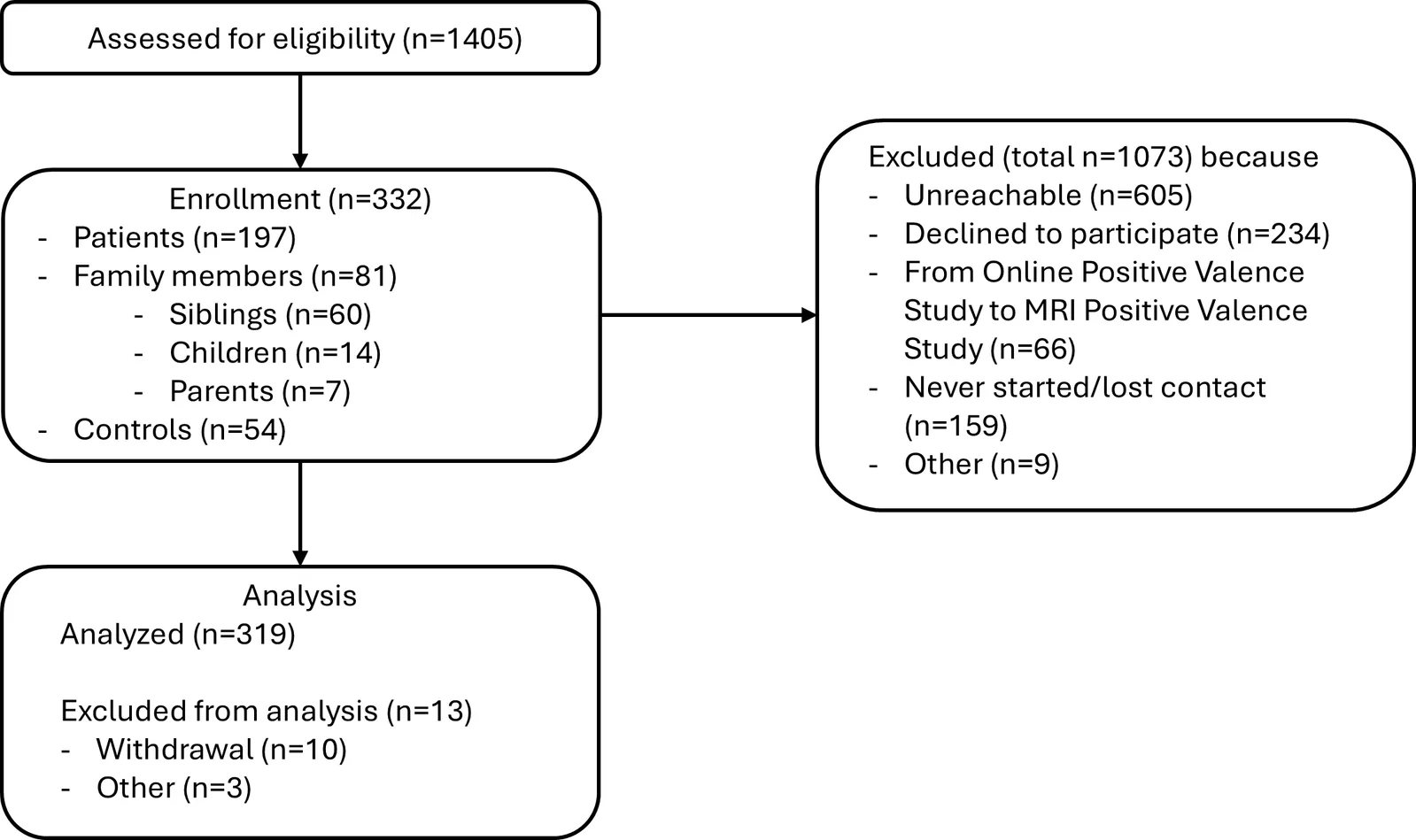
Journal Article Reporting Standards (JARS) participant flowchart.

**Figure 4.**
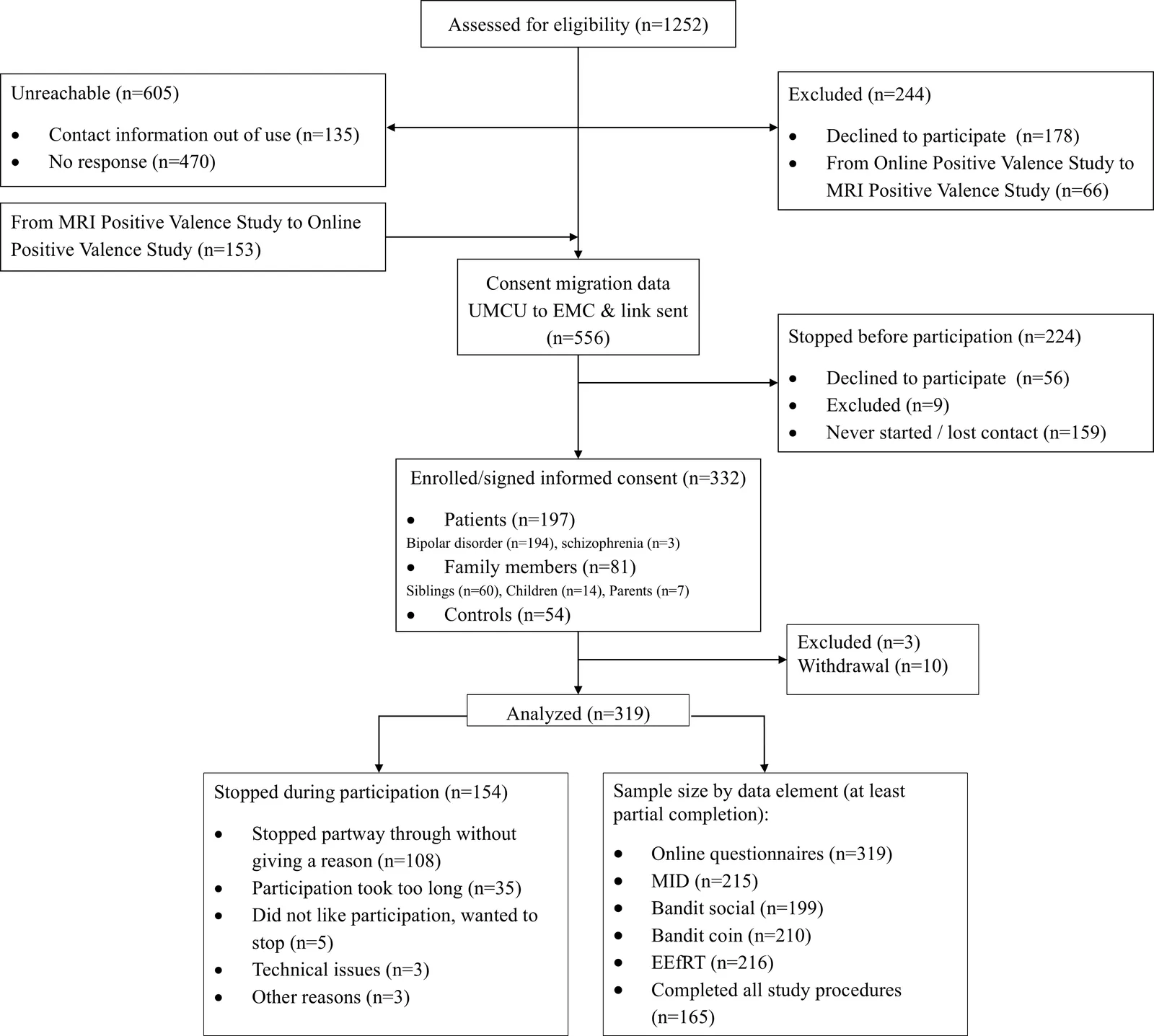
Detailed CONSORT (Consolidated Standards of Reporting Trials) flow diagram of participant flow through each phase of the Online Positive Valence Study, depicting the inflow and outflow of participants throughout recruitment and enrollment. EEfRT=effort expenditure for rewards task; EMC=Erasmus Medical Center; MID=monetary incentive delay; MRI: magnetic resonance imaging; UMCU=University Medical Center Utrecht.

**Figure 5.**
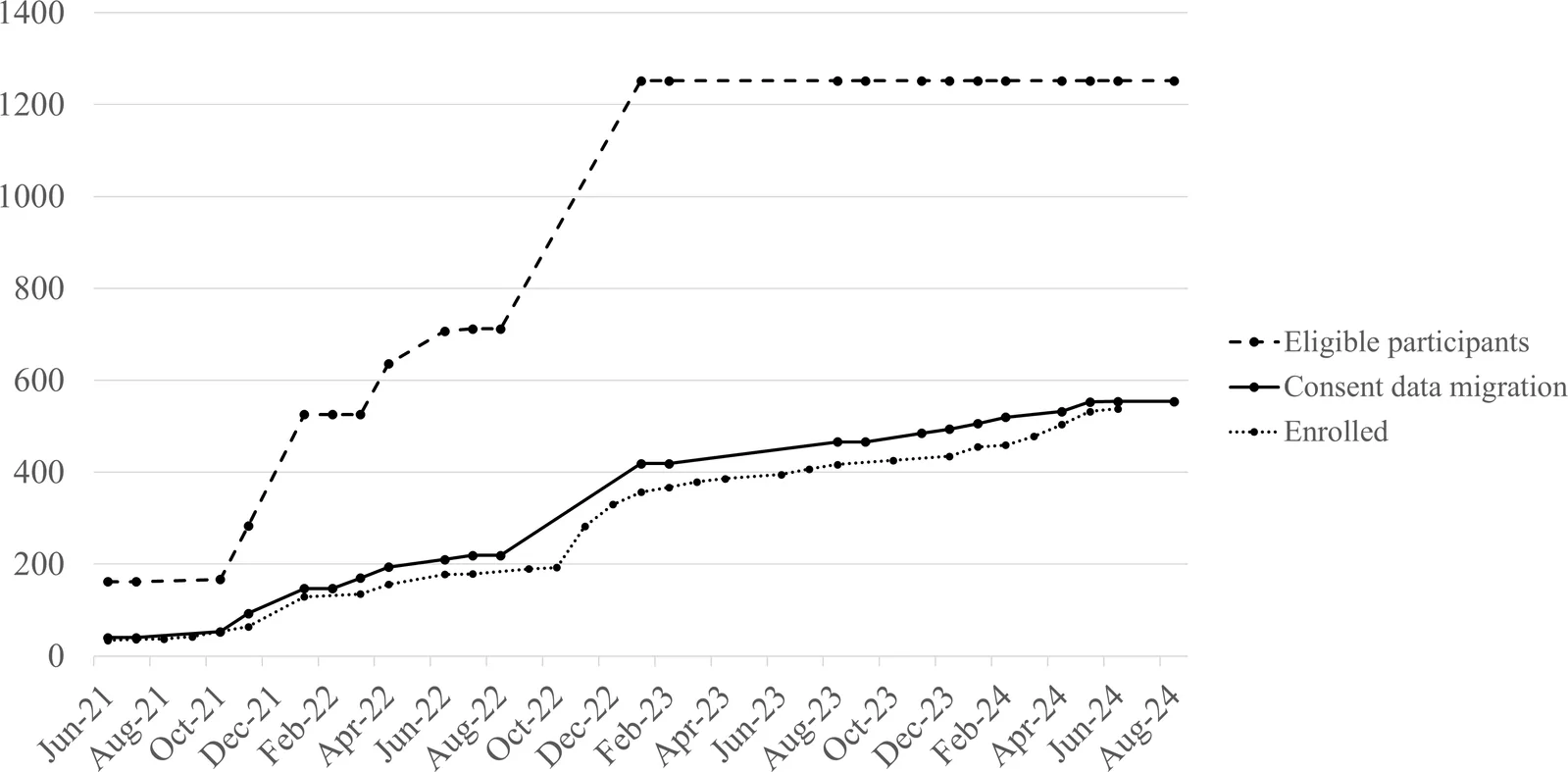
Line chart of cumulative number of participants enrolled over time of the Online Positive Valence Study.

**Figure 6.**
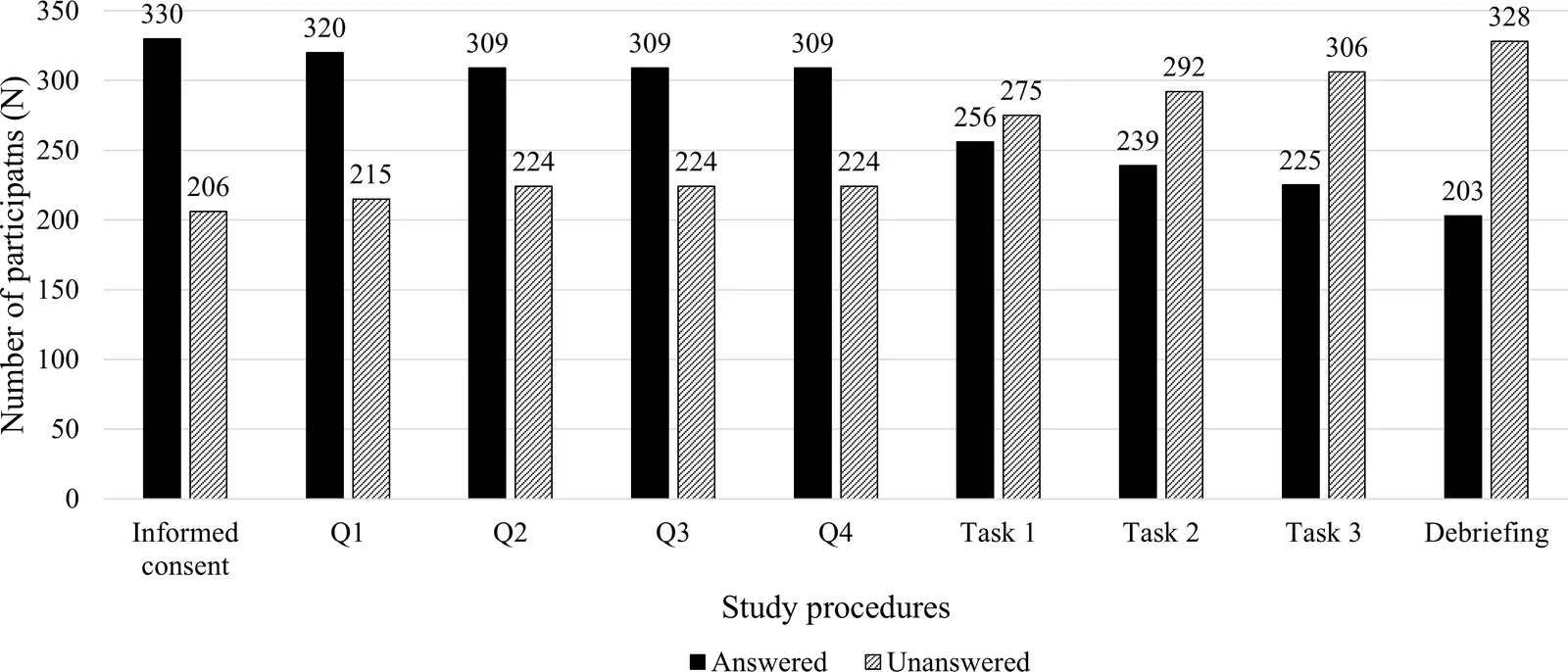
Bar chart of completed and missing responses per study procedure: Q1 to Q4 are online questionnaires split in 4 Limesurvey objects.

### Statistics and Data Analysis

#### Recruitment Strategies

To determine whether the level of personal contact made an improvement to our enrollment, 2 contact strategies were analyzed. First, the difference in enrollment was analyzed between participants who were contacted through emails only and participants whom the research team had spoken to on the phone at least once in addition to the standard emails. We conducted a multinomial logistic regression analysis to examine the association between the 2 modes of contact and the likelihood of response with 3 levels: Yes, No, and No response. Compared with the email-only group, participants who were called were more than 4 times as likely to respond “no” rather than give no response (odds ratio [OR] 4.57, 95% CI 3.12 to 6.70, *P*<.001) and more than twice as likely to respond “yes” rather than give no response (OR 2.75, 95% CI 1.93 to 3.92, *P*<.001). In addition, being called made participants more likely to respond “no” than “yes” (OR 1.67, 95% CI 1.19 to 2.33, *P*<.001; see Table 2).

**Table 2.** Frequency table responses to telephone contact versus no telephone contact.

Contact	Response to participation, n (%)		
	Yes	No	No response
Telephone contact (n=260)	115 (44.2)	91 (35)	54 (20.8)
No telephone contact (n=988)	344 (34.5)	176 (17.6)	478 (47.9)

In addition, logistic regression analysis was used to analyze the effect of a personal salutation in the invitation email on enrollment, measured as the number of signed informed consents (see Table 3). Participants who received an invitation mail with a personal salutation were less likely to sign informed consent compared with participants who received a nonpersonal salutation (OR 0.31, 95% CI 0.20 to 0.47, *P*<.001).

**Table 3.** Frequency table of signed informed consents to a personal versus nonpersonal salutation.

Salutation type	Signed informed consent, n (%)	
	Yes	No
Personal salutation (n=382)	197 (51.6)	185 (48.4)
Nonpersonal salutation (n=163)	126 (77.3)	37 (22.7)

#### Task Performance in the Lab Versus Online

Performance on the reward tasks between participants in the in-lab MRI Study and the Online Study were largely similar (see Tables S3-S6 in Multimedia Appendix 1). Significantly higher reaction times were found during online performance compared with in-lab performance of the MID (*β*=20.43, 95% CI 15.41 to 25.44, SE 2.55; *t*_309.10_=8.02, *P*<.001; η² *P*=.13; see Figure 7 and Table S3 in Multimedia Appendix 1). However, the difference in reaction times between a reward trial and a neutral trial was not significantly different between the 2 contexts (*β*=2.72, 95% CI –0.36 to 5.80*,* SE 1.59; *t*_415_=1.71, *P*=.09; η² *P*<.01). Next, participants in the online group had significantly lower win-stay rates in both Bandit tasks than participants in the in-lab group (Bandit Coin: *β*=–0.55, 95% CI –0.68 to –0.42, SE 0.06; *t*_306.0_=–8.73, *P*<.001; η² *P*=.14; Bandit Social: *β*=–0.65, 95% CI –0.80 to –0.49, SE 0.08; *t*_307.2_=-8.55, *P*<.001; η² *P*=.14; see Figure 8). The win-stay rate was defined as the percentage of times a participant chose the same arm after being rewarded by that arm in the previous trial, averaged across all rounds. All other variables of both Bandit tasks were not statistically different (see Tables S5 and S6 in Multimedia Appendix 1). Performance on the EEfRT did not differ significantly between the online and in-lab groups (see Table S4 in Multimedia Appendix 1).

**Figure 7.**
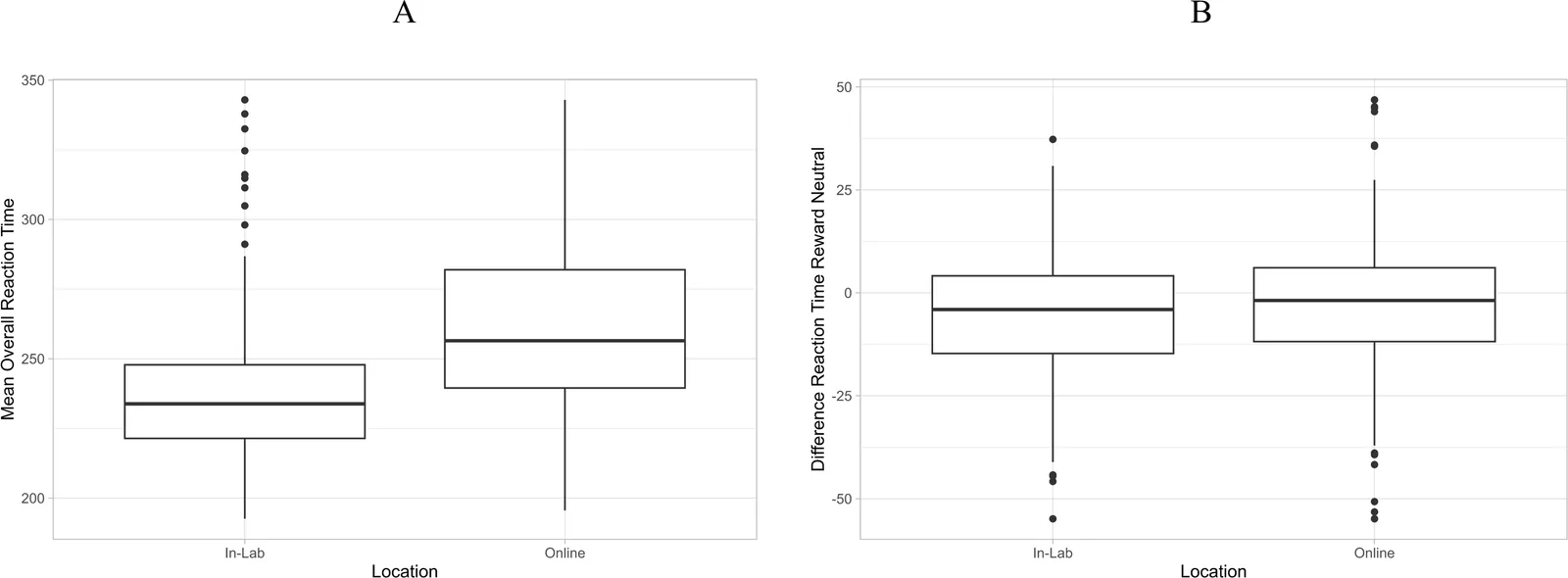
Boxplot of (A) mean overall reaction time and (B) difference in reaction time between reward and neutral trial on the monetary incentive delay (MID) for participants in lab and online; note that reaction time was measured in milliseconds.

**Figure 8.**
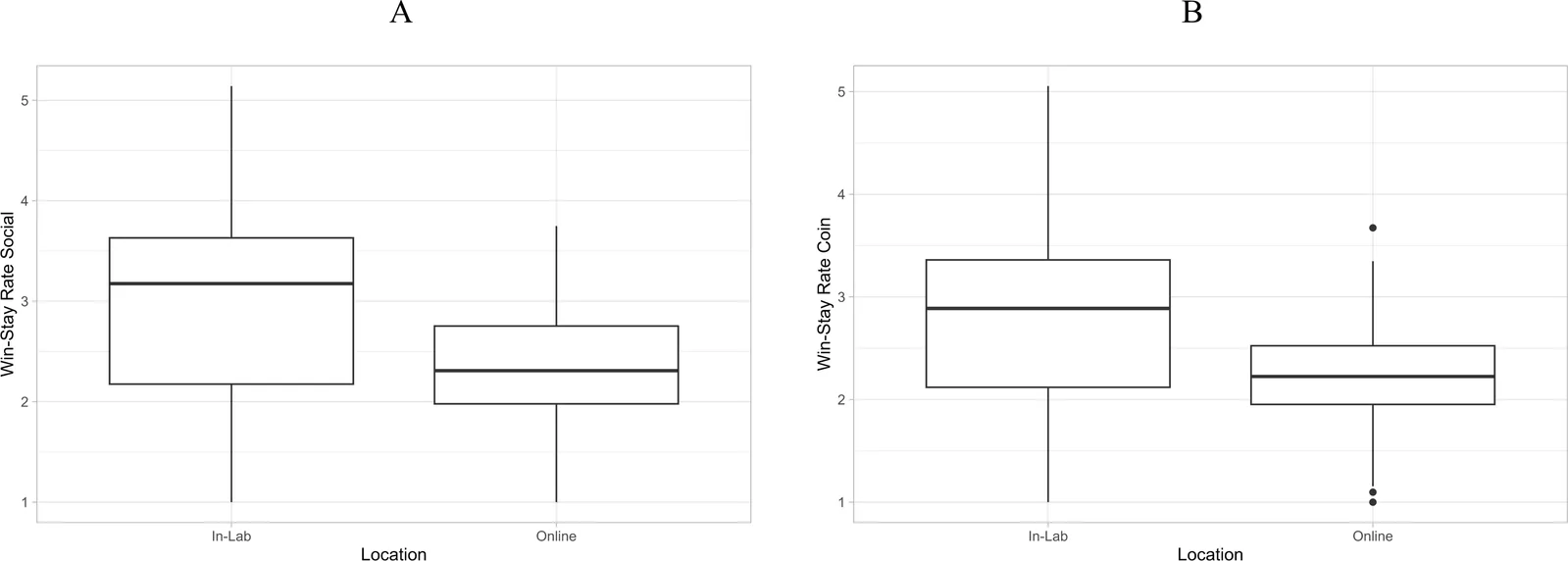
Boxplot of win-stay rates on bandit (A) social and (B) coin for participants in lab and online.

## Discussion

### Principal Findings

This paper attempted to critically evaluate the methodology of the Online Positive Valence Study and our efforts to improve recruitment numbers. The main goal was to support future task-based online studies with our lessons learned and to explore task performance comparability between in-lab and online participant groups. Our main findings were that we have faced a variety of challenges in infrastructure setup, recruitment, time constraints, and participant attrition. We found that calling participants by phone was associated with an increase in responses and enrollment, compared with email contact only. A personal salutation in mails was associated with a decrease in signed informed consent forms. Task performance on 3 reward tasks was similar between participants who carried out the task at home and in lab except for one reward learning task. All analyses comparing recruitment-related strategies and task outcomes were exploratory in nature and not corrected for multiple testing, which should be taken into account when interpreting these findings. An important limitation of our approach is that participants were not randomized to either a study or recruitment strategy. Based on these observations and results of the Online Positive Valence Study integrated with existing literature, we provide 6 lessons we learned from conducting an online task-based study with patients with SMI.

### Take Into Account Legal, Practical, and Psychological Factors in Recruitment and Data Sharing

Once data collection for the Online Study started, it appeared difficult and time-consuming to reach large numbers of eligible participants from existing cohorts (ie, Bipolar Genetics [BiG] and GROUP) due to a number of reasons. First, BiG and GROUP finished in 2015 and in 2013, respectively. Informed consent forms at the time did not ask for explicit consent to be contacted for future research, meaning the team was not allowed to contact all previous participants. Providing explicit consent is a prerequisite under GDPR, which was introduced in 2016. In addition, contact information often turned out to be outdated. Retrieving up-to-date information was a tedious task.

Second, the PVS is conducted on an NIMH grant, while the data collection took place in the Netherlands. It was agreed to upload the data in the National Data Archive in the United States. Data regulations are different between Europe and America, which meant the research team devoted considerable time and effort to ensure privacy regulations adhered to European Union laws while retaining the ability to upload the data to a US-based server. Participants were asked to give explicit consent for data sharing outside of the European Union. If no consent was given, participants could still participate, so the research team did not believe that these issues on privacy regulations and data sharing resulted in lower enrollment of participants.

Third, 3 other independent studies were also recruiting from BiG and GROUP at the same time. An obvious advantage of recruiting from existing cohorts is that a lot of information about the participants is already available from the previous study. However, recruitment from the same pool of participants by 4 independent studies came with many administrative hurdles to ensure participants were not overwhelmed by multiple researchers getting in contact. Additionally, participants were confused whether they had already participated or declined participation before. In August 2023, we decided to also recruit participants outside these 2 cohorts to increase the sample size for the MRI study. Participants declining the MRI Study were offered the Online Study. We reached out to several psychiatry departments in the catchment area, hung up posters in outpatient clinics with a QR code directly leading to information on the experiment, and made use of mailing lists and websites from patient associations. Recruitment could have also benefitted from participants’ own networks, by allowing them to share the QR code with other people. A limitation of recruiting outside the already existing cohorts was that no prior information was available on these participants. We decided not to incorporate more questionnaires as not to further increase the duration of data collection for participants. Indeed, it has been reported that, when the number of online survey items increased, the number of dropouts increased [54]. In addition, almost 80% of respondents in an online survey abort participation after 30 minutes [55]. This aligns with an increase in unanswered parts of data collection over the course of our Online Study (Figure 6). Researchers wanting to conduct an online study should carefully consider the trade-off between collecting large amounts of information and keeping the duration of participation low in order to minimize participant attrition throughout the online study.

### Ensure Sufficient, Well-Trained Personnel

In our hands, recruitment was impacted by the severely understaffed research team. They were unable to call every eligible participant due to time constraints, yet calling participants resulted in more response and thus more enrollment, which has also been found in previous literature [56]. Due to the extensive recruitment process with numerous manual steps (Figure 1), the research assistant was also not able to send out many emails continuously in the time that was allocated to the Online Study. In the recruitment timeline for the Online Study (Figure 5), two clear peaks were shown in the number of recruitment emails sent to eligible participants. In particular, the period immediately after the emails were sent, responses had to be processed, which took a lot of time and effort from the research team. Taken together, the research team did not have enough time and resources to recruit large numbers of participants, and fewer recruitment emails were sent after such peaks.

The researchers of the Online Study noticed that conducting an online behavioral experiment does not require less labor than an in-lab behavioral experiment. Matcham et al [57] conducted a longitudinal study containing online study procedures in people with major depressive disorder and recruited 627 participants, which was well over the target of 600. Oetzman et al [58] shared the lessons learned from this cohort study in a separate paper, where they shared their observations on successful recruitment. They employed 3 full-time research assistants for this study and stated that this team setup was paramount for the success of the study. Their design was elaborate, including various questionnaires, cognitive tasks, speech tasks, and an electronic diary as well as collecting data through a wearable fitness device. The participants were recruited through clinics and online advertisements and met with the research team once, after which they were remotely followed for 6 months up to 3 years. For a more observational study with less data to collect, like our Online Study, we believe it would be realistic to hire at least 2 full-time research assistants. The research assistants would be able to fully commit to the study and to allocate sufficient time for recruitment, troubleshooting, monitoring, and correspondence.

### Carefully Plan a Simple and User-Friendly Infrastructure

To set up the infrastructure for our Online Study, we were forced to use different software for data storage, questionnaire repository, and task execution, which was error-prone. Many reroutes were needed in order to enhance user experience and to ensure privacy regulations. This took up more time than expected, delaying the start of the data collection phase, while during data collection, the complex infrastructure resulted in substantial technological hurdles for participants. For example, participants had to be willing to download external software (Inquisit) on their computer to participate. Grootswagers [59] stated that the core infrastructure of an online study should be browser-based and participants should not have to download external software. They mentioned that several JavaScript modules exist that are tailored to conduct online behavioral experiments, so no programming experience is needed. In addition, all software is generally compatible with web browsers. However, older browsers have different technical limits, making it hard to ensure stability in task presentation between participants [60], which is especially relevant in timed tasks like the MID and could influence task performance. A server like Inquisit allows for a more standardized presentation across participants, since it is not browser-based. That said, online experiments will inevitably have varied task performance due to the fact that participants may use a variety of computer screens, keyboards, computers, and processors.

In this study, online participation was restricted to computers and laptops. This may have created an extra burden, leading to fewer enrolled participants. However, creating a timed task with stable presentation across different user hardware is challenging, as touchscreen and keyboard registration may vary [61]. Validity and implementation of remote mobile-based assessments of cognition in older adults with and without cognitive impairment [62-64] give hope for technological innovations in psychiatric populations. For in-lab or in-clinic mobile-based cognitive assessment through neuropsychological tasks, there is evidence for similar validity compared with paper-and-pencil–based assessments in schizophrenia, major depressive disorder, and bipolar disorder [65-69]. Ultimately, it is a trade-off between better task reliability or a friendly user experience in which the researcher has to decide what to prioritize. Feenstra et al [60] provided guidelines and technical considerations when initiating an online experiment, focusing on technical, contextual, and psychological factors.

### Make Tasks More Interesting to Avoid Attrition

One-half of the participants who started the Online Study did not complete participation, and a clear increase in dropouts was seen when participants started the tasks (Figure 6), which raises concerns on compliance and validity. We believe a possible solution to minimize attrition is through gamification: Adding game-like elements, like a scoring system, to an otherwise mundane cognitive task could enhance engagement [70,71]. A previous qualitative study of a mobile-based cognitive assessment app for patients with schizophrenia noted that patients enjoyed the gamification, the games were challenging, and they disliked repetition [69]. Lumsden et al [72] suggested that careful application of gamification can provide a way to develop scientifically valid experimental cognitive assessments and may thus be applied to boost participant motivation. Apart from having an element to collect points, the tasks in the Online Study did not include gamification and were experienced as boring and tedious, as reported by participants. It could be interesting to see if adding more game-like elements to our reward tasks would decrease attrition. However, if the goal is to make inferences about the measured constructs, future research should first ensure the validity of more heavily gamified experimental cognitive tasks in a separate validation study [73].

### Specific Considerations in Recruitment Related to a Psychiatric Population

Eisner et al [74] noted that, for patients with psychotic disorder, medication side effects and active cognitive, positive, or negative symptoms interfered with engagement in digital mental health interventions. People with psychiatric disorders might refrain from participating in scientific research due to disability inherent to the disorder or due to disorder-related features or stigma [74,75]. It is possible that symptom severity or disease course could have affected the ability of our online participants to complete the Online Study, since patients who did not complete all tasks had more mania symptoms than patients who completed all tasks. Moreover, our attrition analysis showed that participants who did not complete all tasks differed from completers in age and education, suggesting that completion was not random. This suggests that, in the final analytic sample, participants who were younger (and possibly more digitally capable), less symptomatic, or more motivated were over-represented. Future research in better-controlled designs with less attrition is needed to evaluate the validity of online versus in-lab assessments in patients with SMI.

Personal contact is believed to increase response rates in SMI populations [76]. In the successful recruitment strategy of people with major depressive disorder by Matcham et al [57], they invested in personal contact with their participants by calling them throughout recruitment, which might have led to higher enrollment. According to Lynham et al [9], enhancing participant engagement among individuals with a psychotic disorder may require additional support, such as more frequent telephone contact. In our Online Study, calling participants was indeed associated with higher response rates. Despite the majority of the eligible participants declining participation, it was also associated with an increase in participation rates. In addition, personal salutation has been found to increase responses on web surveys in healthy participants [32]. In our sample, a personal salutation was associated with a decrease in responses. Given the nonrandom implementation of personal salutation, this result should not be interpreted as evidence that personalization is detrimental. It could be due to the research team calling participants less often, since they assumed that a personal salutation would lead to an increase in responses. Perhaps disorder-related features, such as mistrust or paranoia, may also have contributed to suspicion toward the personalized salutation in patients. However, it is important to note that the recruitment interventions in our Online Study were not implemented in a structural manner and assignment to recruitment interventions was not randomized. Therefore, the analyses of the effect of the recruitment interventions should be interpreted with extreme caution. Despite these limitations, we propose that a personal approach to maximize recruitment in an online study is strongly advised. Ideally, one would call participants who initially do not respond to personalized emails. It should be taken into account that this approach takes significant effort from the research team.

### Differences in Task Behavior Between the Online Study and In-Lab Study

We compared the outcome variables of the MID, EEfRT, and Bandit between the Online Study participants and In-lab MRI Study participants. The results are exploratory and do not provide conclusions on the validity of remote assessment. On the MID task, online participants were overall significantly slower than in-lab participants, yet the mean difference in reaction time between neutral and reward trials did not differ significantly between the groups. This indicates that, despite being slower online, participants showed a similar decrease in reaction time during trials where they anticipated reward compared with neutral trials. Thus, the manipulation to elicit reward anticipation seems to stay intact when executed in an online environment. For the Bandit Social and Bandit Coin tasks, the win-stay rate was significantly lower in the online group than the in-lab group. The win-stay rate was defined as the percentage of times a participant chose the same arm after being rewarded by that arm in the previous trial, averaged across all rounds. This means that participants in the online group were less inclined to choose the same arm that awarded them in the previous trial than in-lab participants. One possible explanation could be that the online participants were less attentive or concentrated less than the in-lab participants. However, this is generally not supported in the literature [77,78], and the total scores of both Bandits did not differ between the groups (see Tables S5 and S6 in Multimedia Appendix 1), which would be expected when participants are not actively participating in this task. Another possibility is that participants carrying out an online task are more explorative and consciously switch more between arms. This suggests they take on a different reward-learning strategy than in-lab participants. Alternative explanations include differences in task comprehension, environmental distractions, fatigue, device or keyboard differences, or sample composition. However, this could also be a more subconscious process, similar to a finding by Kang and colleagues [78], where they interpreted greater standard deviations of reaction time on go and no-go trials as careless. A common finding is that online participants in both studies exhibited greater variability in their response patterns than the in-lab participants executing the same task. The participant groups did not differ significantly on the other outcome variables of the reward tasks (see Tables S3-S6 in Multimedia Appendix 1), suggesting that task performance on the reward tasks is largely comparable between online and in-lab participants.

Participants were not randomized to the Online Study or MRI Study nor to the recruitment strategies of contact methods and (non)personal salutations. As a result, the groups differed from each other on demographic and clinical variables. This has large implications on the interpretation of the results. Potential confounding variables, such as symptom severity, motivation, or digital literacy, were not controlled for. Results from the comparison of performance should be interpreted cautiously with this limitation in mind.

### Conclusion

The Online Positive Valence Study is among the first to attempt to remotely measure PVS functioning in people with bipolar disorder or schizophrenia, unaffected siblings, and control participants using an online task-based study design. While setting up the study, the research team stumbled upon a variety of obstacles along the way, which led to the study not meeting expectations. This paper provides a critical evaluation of our methodology and shares our lessons learned, with the aim of guiding future researchers in conducting improved online task-based studies to investigate this relevant topic. It is advised to (lesson 1) carefully consider advantages and disadvantages of recruiting from existing cohorts; (lesson 2) hire full-time dedicated personnel and use personal approaches to recruitment, like phone calls; and (lesson 5) adjust recruitment to the target population(s). Furthermore, (lesson 3) participation should be technically as simple as possible using, for example, browser-based presentation of tasks after checking their consistency across the most common browsers. Attrition during the course of the study can be limited even more by (lesson 4) shortening the duration of participation and/or adding gamification. This paper can be used as a practical framework for future researchers to conduct online experiments with reward tasks in psychiatric populations more successfully, so meaningful clinical implications can be made based on their work.

## Supplementary material

10.2196/86998 Multimedia Appendix 1 Supplementary methods, descriptive statistics and symptom measures for online and in-lab participants, interviews and questionnaires, difference statistics, and symptom measure results.

## Abbreviations

ANCOVA: analysis of covariance
ASRM: Altman Self-Rating Mania
BASIS-24: Behavior and Symptom Identification Scale 24
BiG: Bipolar Genetics
DBS: Dutch Bipolar Study
EEfRT: Effort Expenditure for Rewards Task
GDPR: General Data Protection Regulation
GROUP: Genetic Risk and Outcome of Psychosis
JARS: Journal Article Reporting Standards
LLM: linear mixed effects model
MCAR: missing completely at random
MID: monetary incentive delay
MRI: magnetic resononce imaging
NIMH: National Institute of Mental Health
OR: odds ratio
PROMIS: Patient-Reported Outcomes Measurement Information System
PVS: Positive Valence Systems
PVSS: Positive Valence Systems Scale
RDoC: Research Domains Criteria
SMI: severe mental illness
TEPS: Temporary Experience of Pleasure Scale
UMCU: University Medical Center Utrecht
WMO: Wet Mengebonden Onderzoek
