# Supplementary material for: An Online Cross-Sectional Research Domain Criteria (RDoC) Positive Valence Assessment Study With Patients With a Severe Mental Illness: Methodological Evaluation
Source: JMIR Form Res. 2026 Nov 11;10:e86998. doi: 10.2196/86998 (PMC13754185; doi:10.2196/86998)
Supplement: Supplementary file 1 [file formative-v10-e86998-s001.docx]

# **Supplementary Materials**

**Supplementary Methods**

**MRI Study**

Parallel to the Online Positive Valence Study, an MRI study was conducted at the Erasmus Medical Center. As mentioned, participants from DBS and GROUP were selected to participate in this study, in addition to participants that were newly approached, for example through patient organizations, the Erasmus MC in- and outpatient clinic or via the web page of the study. In short, participation in this MRI study entailed one visit to the hospital with a duration of approximately 5.5 hours, including a diagnostic interview, an MRI scan, the same three PVS tasks, the collection of blood and fecal samples as well as assessments of blood pressure, height and weight. After the visit, participants received some additional questionnaires via email. After the visit, participants received 40 euros in e-vouchers and their travel expenses were reimbursed. Apart from the MRI scan and biological measures, the MRI study and the online study are largely similar; however, certain differences should be highlighted. In the MRI study, participants were seated alone in a room and provided with a laptop to complete all three tasks consecutively. A research assistant checked on the participant approximately every 15 minutes to make sure the programme ran smoothly and the participant was comfortable, for example when a participant had to use the bathroom in between tasks, the assistant made sure Inquisit did not shut down by occasionally pressing a key when asked if still present (no tasks or instructions were skipped during this process).

**Participants Characteristics**

Task data was collected of 519 participants, of which 319 participated in the online study and 272 participated in the MRI-study. Descriptive information can be found in Table S1

**Table S1.** Descriptive statistics and symptom measures for participants online and in-lab

|  |  |  | Location of assessment | | | | | | | | |
| --- | --- | --- | --- | --- | --- | --- | --- | --- | --- | --- | --- |
| Descriptives | | | Online | | |  | | | In-Lab | | |
|  |  |  | N | % of total | M | SD |  | N | % of total | M | SD |
| **Sex** | |  |  |  |  |  |  |  |  |  |  |
|  | Male | | 113 | 35.42% |  |  |  | 134 | 49.26% |  |  |
|  | Female | | 206 | 64.58% |  |  |  | 138 | 50.74% |  |  |
| **Age** | |  |  |  | 56.64 | 12.43 |  |  |  | 52.68 | 12.06 |
| **Education level** | | |  |  | 4.01 | 0.99 |  |  |  | 4.01 | 0.85 |
|  | Less than secondary | | 1 | 0.30% |  |  |  | 3 | 1.10% |  |  |
|  | Secondary | | 26 | 8.15% |  |  |  | 24 | 8.82% |  |  |
|  | Vocational tertiary | | 23 | 7.21% |  |  |  | 9 | 3.31% |  |  |
|  | Professional tertiary | | 169 | 52.98% |  |  |  | 122 | 44.86% |  |  |
|  | University | | 81 | 25.40% |  |  |  | 71 | 26.10% |  |  |
|  | Missing | | 19 | 5.96% |  |  |  | 43 | 15.81% |  |  |
| **Research group** | | |  |  |  |  |  |  |  |  |  |
|  | Patient | | 186 | 58.31% |  |  |  | 134 | 49.27% |  |  |
|  | Relative | | 81 | 25.39% |  |  |  | 62 | 22.79% |  |  |
|  | Control | | 52 | 16.30% |  |  |  | 76 | 27.94% |  |  |
| **Symptoms** | | |  |  |  |  |  |  |  |  |  |
|  | Mania | |  |  |  |  |  |  |  |  |  |
|  |  | Patients |  |  | 1.80 | 2.32 |  |  |  | 1.78 | 2.37 |
|  |  | Non-patients |  |  | 1.56 | 2.35 |  |  |  | NA | NA |
|  | Psychosis | |  |  |  |  |  |  |  |  |  |
|  |  | Patients |  |  | 1.44 | 2.27 |  |  |  | 40.10 | 7.97 |
|  |  | Non-patients |  |  | 0.52 | 1.19 |  |  |  | NA | NA |
|  | Depression | |  |  |  |  |  |  |  |  |  |
|  |  | Patients |  |  | 17.80 | 7.44 |  |  |  | 14.30 | 10.30 |
|  |  | Non-patients |  |  | 13.10 | 4.92 |  |  |  | 6.78 | 5.85 |
| *Note*. Depression was assessed using the (PROMIS) in the online study and (IDS) in the MRI-study*.* Psychosis was assessed using the (BASIS24) in the online study and (PANSS) in the MRI-study. | | | | | | | | | | | |

**Measures and covariates**

All interviews and questionnaires used in the Online Positive Valence Study and the in-lab MRI Positive Valence Study can be found in Table S2.

**Table S2.** Interviews and questionnaires used in the online and MRI Positive Valence Study

|  |  | Online Positive Valence Study | MRI Positive Valence Study |
| --- | --- | --- | --- |
| Psychopathology | |  |  |
|  | Altman self-rating mania scale | X | X |
|  | Comprehensive Assessment of Symptoms and History (CASH) | only section 3 (substance use) | X |
|  | Positive and Negative Syndrome Scale (PANSS) |  | X |
|  | Inventory of Depressive Symptomatology |  | X |
|  | Patient-Reported Outcome Measurement Information (PROMIS) (Domain I) | X |  |
|  | Behavior and Symptom Identification Scale (BASIS-24) (4 items) | X |  |
| Positive Valence Systems | |  |  |
|  | Positive Valence Systems Scale (PVSS) | X | X |
|  | Temporary Experience of Pleasure Scale (TEPS) |  | X |
| Medication | |  |  |
|  | Medication questionnaire | X | X |
|  | Alda scale (long-term medication treatment reponse) |  | X |
|  | CRES scale (long-term medication treatment response) |  | X |
| Other | |  |  |
|  | Samengestelde lijst (demographics and disease characteristics) | X | X |
|  | Life Stressor Checklist-Revised (LSC-R) |  | X |
|  | Herstelvragenlijst (episodes or hospitalisations since last assessment) |  | X |

As a measure of mania symptoms in participants with a psychiatric disorder, both studies used the total score of the Altman Self-Rating Mania (ASRM)^1^. For depressive symptoms, the total score of the Inventory of Depressive Symptomatology self-report (IDS-SR)^2^ is used to in all participants of the MRI-study. For the online-study, we used the total score on the 8-item Patient Reported Outcomes Measurement Information System (PROMIS) depression scale^3^ for depressive symptoms. For psychotic symptoms, the total score of the psychosis subscale of the BASIS-24^4^ is used in the online study, and the PANSS positive scale in the MRI-study. For descriptive statistics, we made a distinction between patients with a psychiatric disorder (e.g. schizophrenia and bipolar disorder) and non-patients.

**Supplementary Results**

Results for the MID are summarized in Table S4. Participants who performed the MID online had a significantly higher mean corrected reaction time over all trials compared to participants who performed the MID in-lab. Despite having a higher reaction time overall, there was no significant difference in the differences in reaction time between a reward and a neutral trial between online and in-lab participants.

Results for the EEfRT are in Table S5. There were no differences in total percentage hard task choice and difference in percentage hard task choice for varying probabilities of receiving a reward between the online and in-lab participants.

Results for the Bandit Coin are in Table S6. Participants who performed the Bandit online had a significantly lower win-stay rate compared to participants who performed the Bandit in-lab. There were no significant differences between online and in-lab participants on total points, lose-shift rate and slope best arm.

Results for the Bandit Social are in Table S7. Participants who performed the Bandit online had a significantly lower win-stay rate compared to participants who performed the Bandit in-lab. There were no significant differences between online and in-lab participants on total points, lose-shift rate and slope best arm.

Table S8 shows descriptive statistics and symptom scores of participants who completed all three reward tasks versus participants who did not complete all three reward tasks. Differences were analyzed and significant findings are shared in the main manuscript.

**Table S3.** Summary statistics differences in performance on MID between in-lab versus online participants

|  |  | Estimated coefficient | SE | d.f. | *t* test | *P* value |
| --- | --- | --- | --- | --- | --- | --- |
| **Mean corrected reaction time overall** | |  |  |  |  |  |
|  | Intercept | 223.41 | 9.65 | 93.2 | 23.16 | < .001*** |
|  | Location | 20.43 | 2.55 | 309.1 | 8.02 | < .001*** |
|  | Age | 0.527 | 0.11 | 153.2 | 4.85 | < .001*** |
|  | Sex | 2.97 | 2.56 | 289.9 | 1.16 | 0.248 |
|  | Education level | -3.588 | 1.58 | 70.1 | -2.28 | 0.026* |
| **Difference reaction time reward vs. neutral trial** | |  |  |  |  |  |
|  | Intercept | 12.18 | 5.28 |  | 2.30 | 0.022* |
|  | Location | 2.72 | 1.59 |  | 1.71 | 0.088 |
|  | Age | -0.10 | 0.07 |  | -1.52 | 0.129 |
|  | Sex | -1.51 | 1.58 |  | -0.95 | 0.342 |
|  | Education level | -2.79 | 0.90 |  | -3.10 | 0.002** |

*Note.* MID = Monetary Incentive Delay task; SE = standard error; d.f. = degrees of freedom. (***) < .001; (**) < .010; (*) < .050

**Table S4.** Summary statistics differences in performance on EEfRT between in-lab versus online participants

|  |  | Estimated coefficient | SE | d.f. | *t* test | *P* value |
| --- | --- | --- | --- | --- | --- | --- |
| **Total percentage hard task choice** | |  |  |  |  |  |
|  | Intercept | 49.86 | 7.21 |  | 6.91 | < .001*** |
|  | Location | -3.70 | 2.06 |  | -1.79 | 0.074 |
|  | Age | -0.44 | 0.08 |  | -5.36 | < .001*** |
|  | Sex | -6.77 | 2.08 |  | -3.26 | 0.001** |
|  | Education level | 2.96 | 1.14 |  | 2.60 | 0.009** |
| **Difference percentage hard choice varying probabilities** | |  |  |  |  |  |
|  | Intercept | 27.06 | 7.49 | 411.40 | 3.61 | < .001*** |
|  | Location | 1.85 | 2.14 | 400.84 | 0.86 | 0.388 |
|  | Age | -0.53 | 0.09 | 402.50 | -6.13 | < .001*** |
|  | Sex | -2.20 | 2.16 | 406.16 | -1.02 | 0.309 |
|  | Education level | 4.21 | 1.18 | 411.97 | 3.57 | < .001*** |

*Note.* EEfRT = Effort Expenditure of Rewards Task; SE = standard error; d.f. = degrees of freedom. (***) < .001; (**) < .010; (*) < .050

**Table S5.** Summary statistics difference in performance on Bandit Coin between in-lab versus online participants

|  |  | Estimated coefficient | SE | d.f. | *t* test | *P* value |
| --- | --- | --- | --- | --- | --- | --- |
| **Total points** | |  |  |  |  |  |
|  | Intercept | 179.93 | 4.25 | 407.13 | 42.38 | < .001*** |
|  | Location | -1.93 | 1.21 | 407.68 | -1.59 | 0.112 |
|  | Age | -0.02 | 0.05 | 407.72 | -0.33 | 0.743 |
|  | Sex | -0.57 | 1.21 | 404.79 | -0.47 | 0.636 |
|  | Education level | 1.34 | 0.67 | 403.08 | 1.99 | 0.048* |
| **Win-stay rate** | |  |  |  |  |  |
|  | Intercept | 2.27 | 0.24 | 407.30 | 9.67 | < .001*** |
|  | Location | -0.55 | 0.07 | 404.27 | -8.27 | < .001*** |
|  | Age | 0.01 | 0.01 | 401.12 | 3.22 | 0.001** |
|  | Sex | 0.07 | 0.07 | 406.70 | 1.03 | 0.304 |
|  | Education level | -0.01 | 0.04 | 408.00 | -0.26 | 0.798 |
| **Lose-shift rate** | |  |  |  |  |  |
|  | Intercept | 59.54 | 7.29 | 90.50 | 8.17 | < .001*** |
|  | Location | -2.68 | 1.95 | 305.80 | -1.38 | 0.169 |
|  | Age | -0.21 | 0.07 | 158.10 | -2.77 | 0.006** |
|  | Sex | 2.47 | 1.98 | 301.60 | 1.25 | 0.213 |
|  | Education level | 1.71 | 1.21 | 67.80 | 1.41 | 0.162 |
| **Slope best arm** | |  |  |  |  |  |
|  | Intercept | 0.01 | 0.03 |  | 0.41 | 0.682 |
|  | Location | 0.01 | 0.01 |  | 0.21 | 0.835 |
|  | Age | 0.01 | 0.01 |  | 2.65 | 0.008** |
|  | Sex | -0.01 | 0.01 |  | -0.72 | 0.474 |
|  | Education level | 0.01 | 0.01 |  | 2.38 | 0.018** |

*Note.* SE = standard error; d.f. = degrees of freedom. (***) < .001; (**) < .010; (*) < .050

**Table S6.** Summary statistics difference in performance on Bandit Social between in-lab versus online participants

|  |  | Estimated coefficient | SE | d.f. | *t* test | *P* value |
| --- | --- | --- | --- | --- | --- | --- |
| **Total points** | |  |  |  |  |  |
|  | Intercept | 182.84 | 3.92 | 403.98 | 46.66 | < .001*** |
|  | Location | 0.73 | 1.12 | 402.83 | 0.65 | 0.514 |
|  | Age | -0.07 | 0.04 | 402.85 | -1.64 | 0.102 |
|  | Sex | -1.94 | 1.12 | 403.99 | -1.73 | 0.085 |
|  | Education level | 1.23 | 0.62 | 403.41 | 1.99 | 0.047* |
| **Win-stay rate** | |  |  |  |  |  |
|  | Intercept | 2.78 | 0.28 | 403.46 | 9.95 | < .001*** |
|  | Location | -0.65 | 0.08 | 400.07 | -8.14 | < .001*** |
|  | Age | 0.01 | 0.01 | 399.11 | 1.86 | 0.064 |
|  | Sex | -0.01 | 0.08 | 403.58 | -0.04 | 0.972 |
|  | Education level | -0.03 | 0.04 | 403.88 | -0.72 | 0.469 |
| **Lose-shift rate** | |  |  |  |  |  |
|  | Intercept | 64.66 | 7.51 |  | 8.60 | < .001*** |
|  | Location | -0.75 | 2.00 |  | -0.38 | 0.707 |
|  | Age | -0.24 | 0.08 |  | -2.96 | 0.003** |
|  | Sex | 3.79 | 2.05 |  | 1.85 | 0.066 |
|  | Education level | 0.77 | 1.18 |  | 0.65 | 0.514 |
| **Slope best arm** | |  |  |  |  |  |
|  | Intercept | 0.107 | 0.03 |  | 3.39 | < .001*** |
|  | Location | 0.01 | 0.01 |  | 1.11 | 0.266 |
|  | Age | -0.01 | 0.01 |  | -1.48 | 0.139 |
|  | Sex | -0.01 | 0.01 |  | -0.81 | 0.417 |
|  | Education level | 0.01 | 0.01 |  | 1.24 | 0.217 |

*Note.* SE = standard error; d.f. = degrees of freedom. (***) < .001; (**) < .010; (*) < .050

**Table S7.** Descriptive statistics and symptom measures for online participants who completed all three reward tasks and online participants who did not complete all three reward tasks.

|  | Task completion | | | | | | | | | |
| --- | --- | --- | --- | --- | --- | --- | --- | --- | --- | --- |
| Descriptives |  | Completed all tasks (N = 148) | | | |  | Did not complete all tasks (N = 171) | | | |
|  |  | N | % of total | M | SD/Range |  | N | % of total | M | SD/Range |
| **Sex** |  |  |  |  |  |  |  |  |  |  |
| Female |  | 96 | 64.86% |  |  |  | 110 | 64.33% |  |  |
| Male |  | 52 | 35.14% |  |  |  | 61 | 35.67% |  |  |
| **Age** |  |  |  | 53.44 | 25.4-81.0 |  |  |  | 59.4 | 28.9-88.9 |
| **Education level** |  |  |  |  |  |  |  |  |  |  |
| Less than secondary |  | 0 | 0.00% |  |  |  | 1 | 0.58% |  |  |
| Secondary |  | 7 | 4.73% |  |  |  | 19 | 11.11% |  |  |
| Vocational tertiary |  | 8 | 5.41% |  |  |  | 15 | 8.77% |  |  |
| Professional tertiary |  | 84 | 56.75% |  |  |  | 85 | 49.72% |  |  |
| University |  | 45 | 30.41% |  |  |  | 36 | 21.05% |  |  |
| Missing |  | 4 | 2.70% |  |  |  | 15 | 8.77% |  |  |
| **Symptoms** |  |  |  |  |  |  |  |  |  |  |
| Mania |  |  |  |  |  |  |  |  |  |  |
| Patients |  |  |  | 1.33 | 1.84 |  |  |  | 2.05 | 2.55 |
| Non-patients |  |  |  | 1.57 | 2.18 |  |  |  | 1.61 | 2.58 |
| Psychosis |  |  |  |  |  |  |  |  |  |  |
| Patients |  |  |  | 1.28 | 2.24 |  |  |  | 1.61 | 2.32 |
| Non-patients |  |  |  | 0.28 | 0.67 |  |  |  | 0.80 | 1.56 |
| Depression |  |  |  |  |  |  |  |  |  |  |
| Patients |  |  |  | 18.56 | 7.52 |  |  |  | 17.57 | 7.34 |
| Non-patients |  |  |  | 12.72 | 4.54 |  |  |  | 13.61 | 5.41 |

Supplement references

1. Altman EG, Hedeker D, Peterson JL, Davis JM. The Altman self-rating mania scale. *Biol Psychiatry*. 1997;42(10):948-955.
2. Rush AJ, Carmody T, Reimitz PE. The Inventory of Depressive Symptomatology (IDS): clinician (IDS-C) and self-report (IDS-SR) ratings of depressive symptoms. *Int J Methods Psychiatr Res*. 2000;9(2):45-59.
3. Cameron IM, Cunningham L, Crawford JR, et al. Psychometric properties of the BASIS-24© (behaviour and symptom identification scale–revised) mental health outcome measure. *Int J Psychiatry Clin Pract*. 2007;11(1):36-43.
4. Pilkonis PA, Choi SW, Reise SP, Stover AM, Riley WT, Cella D; PROMIS Cooperative Group. Item banks for measuring emotional distress from the Patient-Reported Outcomes Measurement Information System (PROMIS®): depression, anxiety, and anger. *Assessment*. 2011;18(3):263-283.
